# International Medical Graduate Advising Recommendations From the Council of Residency Directors in Emergency Medicine Advising Student Committee

**DOI:** 10.7759/cureus.10130

**Published:** 2020-08-30

**Authors:** Xiao Chi Zhang, Zachary J Jarou, Dimitry Danovich, Adam R Kellogg, Lucienne Lutfy-Clayton, Adam Kenney, Mary Ann Edens, Emily Hillman

**Affiliations:** 1 Emergency Medicine, Thomas Jefferson University, Philadelphia, USA; 2 Emergency Medicine, University of Chicago Medical Center, Chicago, USA; 3 Emergency Medicine, New York University Langone Health, New York City, USA; 4 Emergency Medicine, Bellevue Hospital Center, New York City, USA; 5 Emergency Medicine, Baystate Medical Center, Springfield, USA; 6 Emergency Medicine, University of Massachusetts Medical School-Baystate Medical Center, Springfield, USA; 7 Emergency Medicine, Rutgers University, Newark, USA; 8 Emergency Medicine, Louisiana State University Health Science Center Shreveport, Shreveport, USA; 9 Emergency Medicine, Truman Medical Center - University of Missouri-Kansas City School of Medicine, Kansas City, USA

**Keywords:** advising, emergency medicine, international medical graduate

## Abstract

International Medical Graduate (IMG) physicians applying to residency training programs in a country different from where they completed medical school, bring beneficial diversity to a training program, but also face significant challenges matching into an Accreditation Council for Graduate Medical Education (ACGME)-accredited residency program. Despite the growing number of IMG applications in Emergency Medicine (EM), there is a paucity of targeted recommendations for IMG applicants. As a result, the Council of Residency Directors (CORD) Advising Students Committee in EM (ASC-EM) created a dedicated IMG Advising Team to create a set of evidence-based advising recommendations based on longitudinal data from the National Residency Match Program (NRMP) and information collected from EM program directors and clerkship directors. IMG applicants should obtain at least two EM standardized letters of evaluation (SLOEs), review IMG matched percentages for programs-of-interest, analyze their objective scores with the previous matched cohorts, and rank at least 12 programs to maximize their chances of matching into EM.

## Introduction

International Medical Graduates (IMG) can offer a wealth of medical knowledge, as well as cultural and academic diversity to a domestic emergency medicine (EM) residency program. While IMG applicants, both United States (U.S.) citizen (U.S. IMG) and non-U.S. citizen IMG (non-U.S. IMG) comprise only 5% of the entire EM application pool, they are the third-largest group, second only to U.S. allopathic seniors (U.S. senior, 71%) and osteopathic (D.O., 21%) applicants [1]. Despite the value of their unique perspectives and skills, IMG applicants face the challenge of being “screened” out early during the application process [2]. Non-U.S. IMG applicants must find institutions that will sponsor visas during their training and participate in a rigorous certification process through the Educational Commission for Foreign Medical Graduates (ECFMG).

Historically, the number of available EM residency positions has exceeded the number of U.S. senior applicants, allowing IMG, and other “non-traditional” applicants to fill in these gaps [3]. However, recent data suggest that the number of IMG applications has tapered off in the last few years despite the fairly stable rate of EM spots filled by U.S. seniors [1]. In 2018, there were 2,278 emergency medicine postgraduate year 1 (PGY-1) positions with 2,901 total applicants; EM had a 99.4% fill rate with only 13 unfilled positions [1]. Approximately 71% of those spots were matched to U.S. seniors and 21% were filled by osteopathic applicants. In the past five years, only 4.2% of available EM positions were filled by U.S. citizen IMG and 1.7% by non-U.S. citizen IMG on average [1].

In 2016, the Council of Residency Directors (CORD) Advising Students Committee in EM (ASC-EM) - formerly known as the Student Advising Task Force - published the Student Advising Recommendations and resources to guide prospective EM applicants and their advisors [4]. While these recommendations were generated based on the available literature, member opinion, and existing advising resources, the authors recognized special applicant population groups, such as IMG, require tailored guidelines beyond the traditional prospective EM applicant recommendations. Given the paucity of targeted recommendations for IMG applicants, the CORD ASC-EM formed an IMG Advising Team for generating advising recommendations that highlight the existing application hurdles for IMG applicants and compile evidence-based guidelines for both the students and their advisors. Where a paucity of objective evidence exists, expert opinion from the CORD ASC-EM was obtained. Our primary objective is to provide recommendations from the ASC-EM IMG Advising Team to maximize the IMG applicants’ chances of matching into an Accreditation Council for Graduate Medical Education (ACGME) EM residency program.

## Materials and methods

Investigators from the CORD ASC-EM identified best-practice advising information through the collation of available literature, existing advising resources, members’ opinions, and the National Resident Matching Program (NRMP) data collected from 2009 to 2018, specifically focusing on the admission patterns for IMG applicants in comparison to U.S. senior applicants. Investigators also independently interviewed various EM Program Directors (PDs), Assistant Program Directors (APDs), and Clerkship Directors (CDs) during national CORD conferences between 2015 and 2016 on their perception of hiring IMG residents. Drafted recommendations were then made available for real-time commentary to the entire ASC-EM, composed of EM faculty and residents across the country as part of the modified Delphi review process. Furthermore, the investigators sought out additional feedback from the CORD members through listserve and blog posts for their perspectives on IMG applicants. All comments were reviewed and addressed summatively by the investigators and commentators via digital communications before convening together to make the final list of recommendations.

## Results

Upon reviewing all available medical literature and collective data from on-line resources through numerous iterative consensus national CORD conference meetings, the CORD ASC-EM IMG Advising Team developed six recommendations as a task force, soliciting comments, revision, and approval as part of a collaborative effort to improve the advising of IMG applicants. The recommendations, as listed below, were designed to serve as a single, referenceable document for IMG applicants, and contained both general and targeted guidelines for a successful EM application process. 

**Figure 1:**
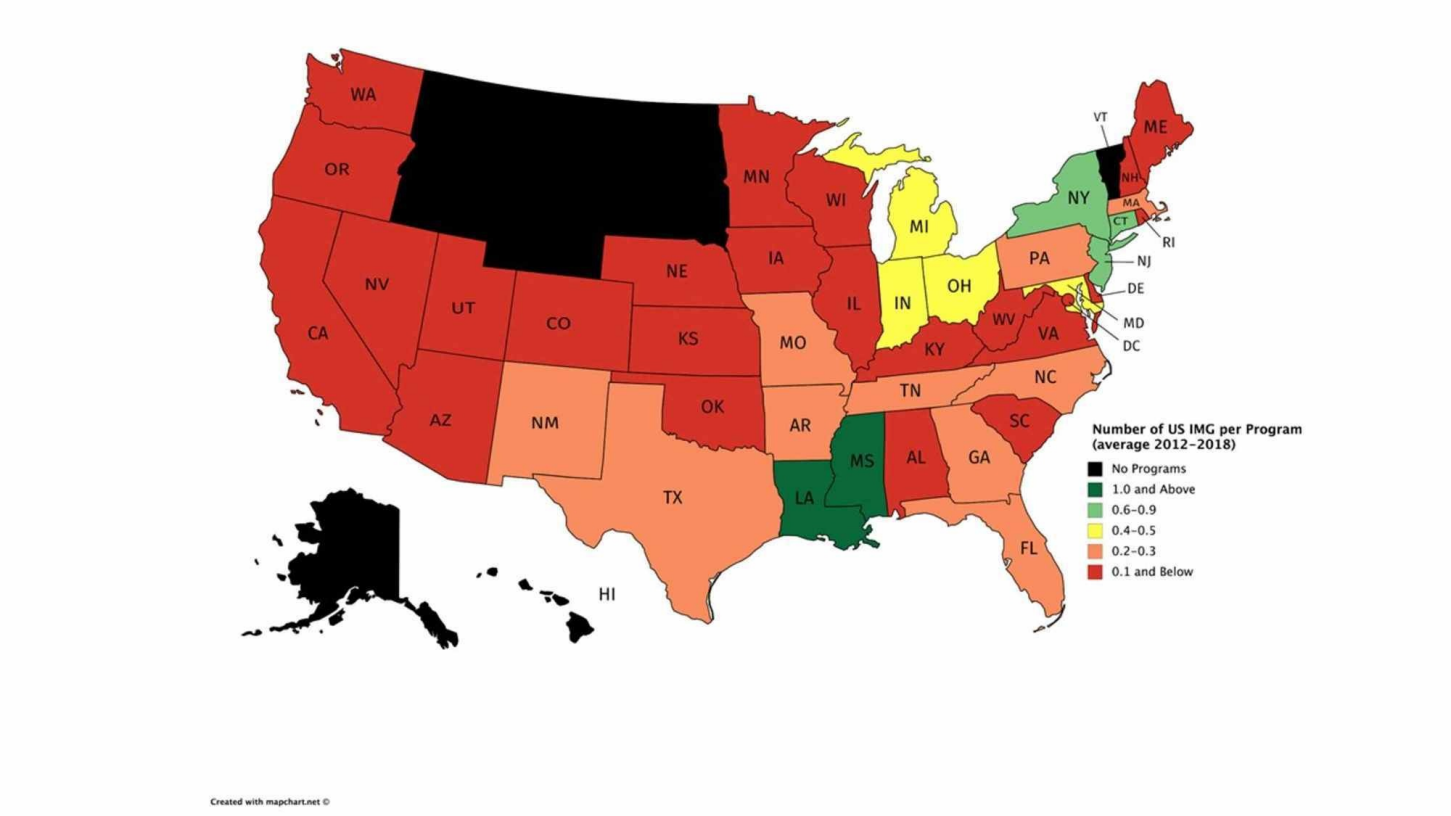
Average number of matched United States International Medical Graduates per Accreditation Council for Graduate Medical Education-accredited residency program per year by state from 2012 to 2016 Source: [5]

1. Identify the Challenges: While U.S. IMG and non-U.S. IMG are slightly different cohorts, it is challenging for EM program directors to be familiar with the myriad of international medical schools; for this reason, IMG may be considered higher academic risks and held at a higher level of scrutiny. According to data obtained via the CORD listserv in 2014, more than half of all residency programs use screening filters; 70% of programs reported screening to exclude non-U.S. IMG, and 45% reported screening to exclude U.S. IMG [2]. Additionally, hiring IMG residents may result in additional financial burdens to a residency program as some medical institutions only sponsor certain types of visas, and for others, the department has to fund the position. As a result of these challenges, IMG need to demonstrate objective mastery of the medical arts via top grades, strong letters of evaluations, exemplary United States Medical Licensing Exam (USMLE) scores, and a robust CV in order to secure an EM residency position.

2. Emergency Medicine Rotations: IMG applicants should complete at least two EM clerkship rotations at institutions with an existing EM residency. Multiple rotations enable applicants to be exposed to a variety of practice patterns, locations, and program designs while allowing program directors to better assess an applicant’s progress from each clinical environment. Summer months (July, August, and September) are the most popular months for EM rotations, although many hospitals often reserve these positions for students from their affiliated medical school. In order to find viable rotation options, IMG applicants can review their institution’s record of where their students have rotated and matched in the previous years, as well as directly contact the departments where they are interested in rotating. Students can also consider using web-tools like Emergency Medicine Residents’ Association (EMRA) Match Residency and Clerkship. This interactive software allows prospective applicants to search for clerkship positions and residency programs [5]. Users can filter programs that accept a high percentage of IMG residents and search for clerkships that consider IMG students. Securing EM rotations may be challenging, as only 32% (48/151) of available clerkships with complete data on EMRA Match accept IMG applicants [5]. 

3. Residency Program Selection: Recent geographic trend analysis from the 2018 NRMP data showed higher U.S. IMG matched percentages in the northeastern and southeastern U.S., with higher matching rates of U.S. IMG residents (Figure 1) [6]. Although these data trends do not reflect the intrinsic values from IMG students, IMG applicants seeking to pursue an EM residency in specific US geographic locations may benefit from reviewing these geographic trends to maximize their match rate. 

4. Standardized Letter of Evaluation (SLOE): One of the most important goals as an EM applicant is to secure an SLOE during an away rotation in the emergency department. A SLOE is cited by EM program directors as the most important factor in their decision to offer an interview and cited as the top five factors when assembling their rank lists, rivaling the importance of USMLE performance [7]. As a result, in order to reflect their EM qualifications, IMG should rotate through EM clerkships with associated EM residency programs with the goal of obtaining two SLOEs. While rotations at non-academic emergency departments may be easier to obtain, the letters of recommendation from these institutions do not follow the standard objective; in the absence of an SLOE, these letters will not carry sufficient weight to assist the IMG applicant in obtaining interviews.

5. USMLE Step 1 and Step 2 Clinical Knowledge (CK) Performance: Applicants are strongly encouraged to have both USMLE Step 1 and Step 2 CK results available when the Electronic Residency Application Service (ERAS) opens. In the 2018 match, the mean Step 1 and 2 scores for matched U.S. IMG were 232 and 241 respectively and for non-U.S. IMG were 229 and 234, respectively [8]. The average for US allopathic seniors who matched was 233 and 247 respectively [9]. IMG applicants with either score below 220 (the average Step 1 score for unmatched US allopathic seniors) should consider consulting with their faculty mentors and consider applying to another specialty simultaneously as a parallel plan [7,8]. 

6. Interviews and Rank List: Although no number of interviews guarantees a 100% match rate, the standard recommendation for US IMG applicants is to apply to enough EM residencies in order to rank at least 12 programs [7]. For non-US IMG applicants, the rank list needs to be even longer. According to NRMP data, a longer rank list correlated with higher match rate for IMG applicants: matched U.S. and Non-U.S. IMG have higher mean rank lists (6.7 and 4.1) than their unmatched counterparts (2.2 and 1.8) [8]. During the interview, IMG applicants should reflect on their unique academic and cultural background, and highlight their unique service, leadership skills, and any applicable research.

## Discussion

To date, this study represents the first evidenced-based recommendation with the emergency medicine leadership consensus for IMG students applying to emergency medicine residency. These recommendations were designed for the typical IMG applicant during normal application seasons; individual IMG applicants should be encouraged to review these recommendations to tailor to their specific needs and circumstances. While these recommendations may provide a helpful guiding framework to non-IMG applicants, they were synthesized based on a careful assessment of NRMP data of IMG applicants, evaluating for adjustable, critical elements of their application that can improve their chances of matching.

Based on the above recommendations, the CORD ASC-EM has developed an online standalone resource for IMG applicants with key recommendations made available on *The Vocal CORD* blog, which has more than 2,500 followers and is used as a reference guide by the entire CORD community [10]. The IMG recommendations post was viewed over 800 times in less than one year. IMG and their advisors are encouraged to utilize these recommendations as general guidelines; however, each applicant still needs an individualized approach. Similar to what was done with other CORD ASC-EM advising resources, the resource is endorsed by CORD, CDEM (Clerkship Directors in Emergency Medicine), EMRA, and the AAEM (American Academy of Emergency Medicine). To date, it has disseminated via multiple avenues to students and their advisors.

While these recommendations were established utilizing the most up-to-date NRMP data, consensus expert opinions from EM program and clerkship directors, the investigators recognize that there is a still limited scope of resources and lack of published data on IMG. Furthermore, the investigators acknowledge that even though there are demographic differences between U.S. IMG and non-U.S. IMG applicants, there is insufficient data to create a separate CORD ASC-EM recommendation distinguishing both subpopulations. Furthermore, the geographic trend of matched-IMG applicants per EM residency [Figure 1] may obscure individual programs in states with numerous residencies that accept high numbers of IMG. The investigators acknowledge that recommendations informed by experience and expertise remain subject to inherent prejudice and bias. Finally, the authors understand that due to unforeseen circumstances of the novel coronavirus disease 2019 (COVID-19) global pandemic, these recommendations may temporarily not apply as many institutions are restricting visiting students and in-person interactions with faculty [11]. However, we are confident that as the situation resolves, and medical schools and hospitals re-equilibrate, these recommendations will once again apply.

## Conclusions

Based on CORD survey results and available NRMP data (as of 2018), the CORD ASC-EM created a list of evidence-based recommendations for IMG applicants applying to ACGME EM residency programs to maximize their chance of matching. IMG applicants should recognize the inherent challenges in applying to EM, consider completing at least two EM clerkships (if applicable) with the goals of obtaining SLOEs, review IMG matched percentages for programs-of-interest, compare their examination scores with the previous matched cohorts, and rank at least 12 programs (or more) to maximize their chances of matching into EM. The CORD ASC-EM IMG Advising Team believes this advising resource will form a foundation for IMG and their advisors to better understand the EM application process.
